# SBUF-SMUF: On the path to the optimum hemofiltration technique in pediatric cardiopulmonary bypass: A randomized clinical trial

**DOI:** 10.1051/ject/2026020

**Published:** 2026-09-15

**Authors:** Mehrtash Shadmehr, Babar Ali, Rosa Abdollahzadeh, Khalil Zarrabi, Hamid Gerami, Salman Parvaiz Butt

**Affiliations:** 1 Department of Anesthesiology, School of Medicine, Shiraz University of Medical Sciences PO BOX 71348-14336 Shiraz Iran; 2 Department of Cardiology and Cardiac Perfusion, Khyber Medical University Peshawar PO BOX 25160 Pakistan; 3 Department of Perfusion, School of Medicine, Isfahan University of Medical Science PO BOX 7346-81746 Isfahan Iran; 4 Department of Cardiovascular Surgery, School of Medicine, Shiraz University of Medical Science PO BOX 71348-14336 Shiraz Iran; 5 Shiraz University of Medical Sciences PO BOX 71348-14336 Shiraz Iran; 6 Perfusion Services, Heart Vascular and Thoracic Institute Cleveland Clinic Abu Dhabi PO BOX 112412 United Arab Emirates

**Keywords:** Pediatric cardiac surgery, Cardiopulmonary bypass, Hemofiltration, SBUF-SMUF, Ultrafiltration techniques

## Abstract

*Background:* Hemofiltration during pediatric cardiopulmonary bypass (CPB) is essential to mitigate hemodilution, inflammatory responses, and postoperative complications. Conventional ultrafiltration (CUF) is widely practiced, but novel methods such as subzero balanced ultrafiltration combined with simple modified ultrafiltration (SBUF-SMUF) may offer superior outcomes. *Objective:* To compare the clinical efficacy and safety of CUF versus SBUF-SMUF in pediatric patients undergoing open-heart surgery. *Methods:* In this prospective randomized, outcome-assessor-blinded clinical trial, 80 pediatric patients with congenital heart disease were allocated to either CUF (*n* = 40) or SBUF-SMUF (*n* = 40) during CPB. Baseline demographics, intraoperative characteristics, fluid balance, blood product utilization, and postoperative outcomes were assessed. Statistical analysis was conducted using independent *t*-tests, Chi-square tests, and Fisher’s exact tests. Statistical significance was defined as a two-tailed *p*-Value of less than 0.05. *Results:* Demographic and preoperative variables were comparable between groups. Mean fluid balance was significantly positive in the CUF group (+192.1 ± 178.8 mL) but negative in the SBUF-SMUF group (–105.0 ± 78.9 mL, *p* < 0.001). The SBUF-SMUF group required significantly less intraoperative PRBC (141.7 ± 70.2 vs. 261.0 ± 68.8 mL, *p* < 0.001) and postoperative FFP transfusion (2% vs. 10%, *p* = 0.020). Post-bypass hematocrit levels were higher (31.2% vs. 27.3%, *p* = 0.001) and extubation occurred earlier in the SBUF-SMUF group (1.05 ± 1.03 vs. 2.27 ± 2.37 days, *p* = 0.006). No significant differences were observed in potassium levels or incidence of acute kidney injury. *Conclusion:* The SBUF-SMUF technique is safe, effective, and superior to CUF in optimizing fluid balance, reducing blood product utilization, and facilitating earlier extubation in pediatric cardiac surgery. No additional risk of oliguria or electrolyte imbalance was observed compared to CUF. Its simplicity and reproducibility support its consideration as a standard approach in pediatric CPB.

## Introduction

Advancements in cardiopulmonary bypass (CPB) technology have enabled surgeons to perform complex procedures effectively, including the repair of heart defects, replacement of malfunctioning heart valves, creation of bypasses for blocked coronary arteries, and heart transplants [1].

Heart defects are among the most common congenital anomalies, affecting approximately 1% of all children born in the United States, which translates to around 40,000 cases annually [2]. Notably, one in four children with congenital heart disease (CHD) requires surgical intervention within their first year of life [3]. Given the complexity of pediatric cardiac conditions, the specific location of defects, and the high level of accuracy required during repairs, CPB has become an essential component in surgeries for CHD [4].

While the CPB process is critical for enabling these surgeries, it can also lead to various physiological changes. One major concern is the activation of systemic pathways that can impact multiple organ systems. Factors such as blood contact with artificial surfaces, the volume of priming fluid, temperature variations, and non-pulsatile blood flow contribute to these responses. Such changes are known to affect cytokine activity and trigger the complement system, potentially leading to systemic effects [5].

Another challenge associated with CPB is hemodilution, which occurs due to the use of crystalloid solutions to prime the CPB circuit. Hemodilution can reduce osmotic colloid pressure, diminish oxygen-carrying capacity due to lower red blood cell volume, and lead to complications such as reduced renal perfusion, vasodilation, and coagulopathy. These effects are particularly concerning in pediatric patients due to the relatively small difference between their blood volume and the necessary priming fluid [6].

To address these issues, CPB circuits are equipped with hemoconcentrators—devices designed to reduce plasma fluid volume. These devices help eliminate excess electrolytes, remove waste products, and prevent dilutional coagulopathy [7].

Ultrafiltration techniques, which optimize CPB, fall into two categories: continuous and intermittent methods. Continuous ultrafiltration is performed over an extended period, while intermittent techniques include conventional ultrafiltration (CUF), modified ultrafiltration (MUF), and simple-modified ultrafiltration (SMUF), conducted in shorter bursts. Continuous methods also include zero-balanced ultrafiltration (Z-BUF) and subzero-balanced ultrafiltration (SBUF), each with distinct benefits [8].

The ideal approach would integrate the advantages of all ultrafiltration methods while prioritizing ease of use and safety. A novel technique known as subzero balanced ultrafiltration–simple modified ultrafiltration (SBUF-SMUF) was introduced by Bierer et al. in 2022. Although the technique and its circuit assembly have been thoroughly described, its clinical outcomes and statistical comparisons are yet to be fully evaluated [9].

This study aims to compare conventional ultrafiltration with a novel technique in pediatric cardiac surgeries to determine which method offers greater benefits for patient recovery.

The rationale for comparing conventional ultrafiltration with a novel technique in pediatric cardiac surgeries is grounded in the pursuit of improved patient outcomes and recovery times. Pediatric patients, particularly those undergoing cardiac procedures, often face unique challenges due to their smaller size and different physiology compared to adults.

### Trial design

This study was a randomized, parallel-group, outcome-assessor-blinded clinical trial performed in Shahid Fagihi Hospital, Shiraz, Iran, from July 2023 to September 2024.

### Participants

**Figure 1:**
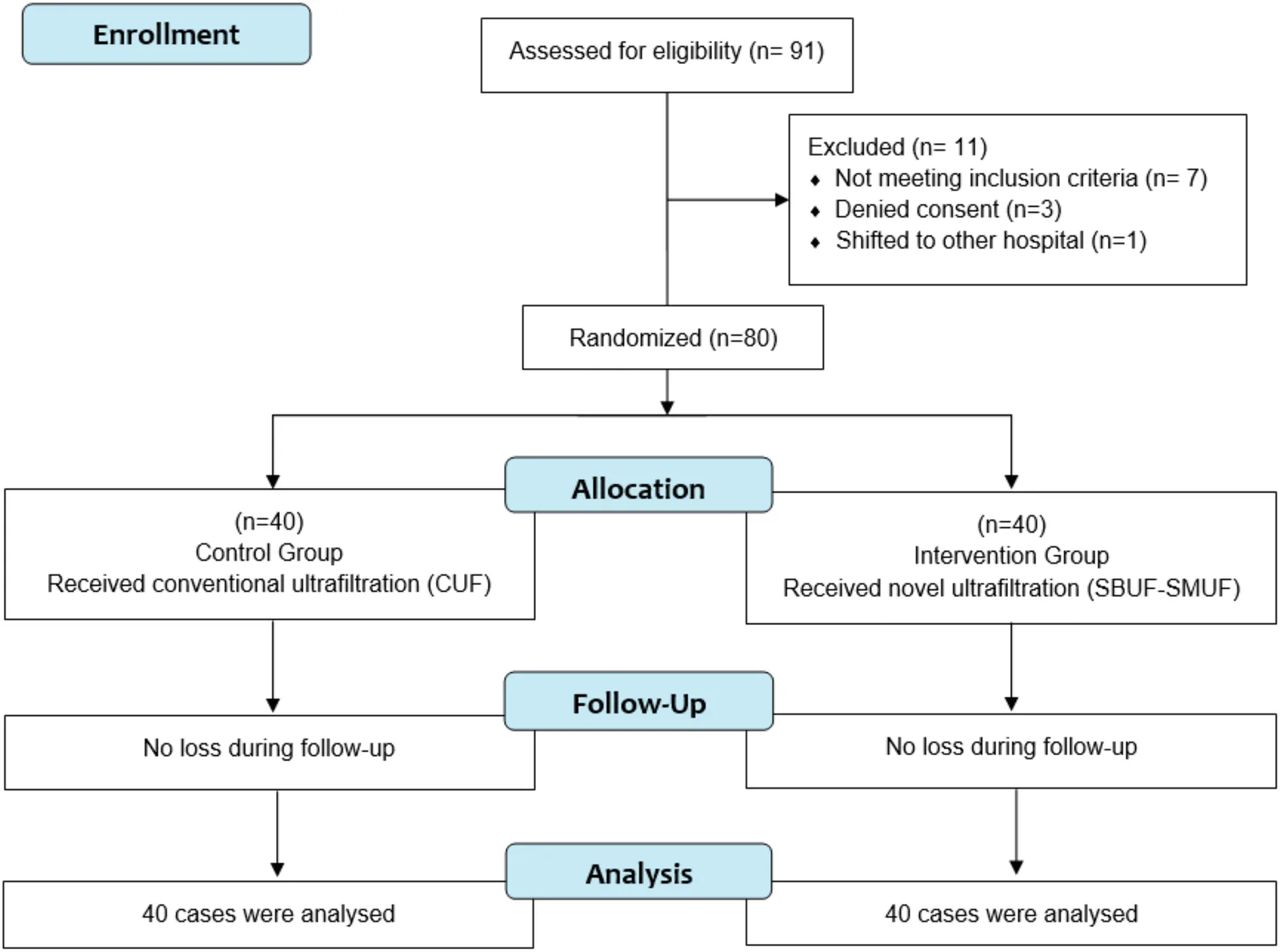
Flow chart of participant selection and group allocation.

A total of 91 patients were initially screened. Out of these, 80 patients were randomly assigned to two groups: the Control Group (*n* = 40) and the Intervention Group (*n* = 40) (Figure 1). Patients were included based on the following criteria:

- **Inclusion criteria**: Patients with congenital heart disease who were candidates for open-heart surgery, aged 1 month to 18 years, weighing 4 to 40 kg, with a pre-bypass hematocrit level below 45%, and a pump time of at least 45 min.
- **Exclusion criteria**: Patients diagnosed with transposition of great arteries (TGA) (to maintain homogeneity, as TGA often requires unique surgical strategies such as arterial switch operation with differing ultrafiltration needs), those with a history of premature birth, and those whose parents did not provide informed consent for participation.

### Interventions:

**Figure 2:**
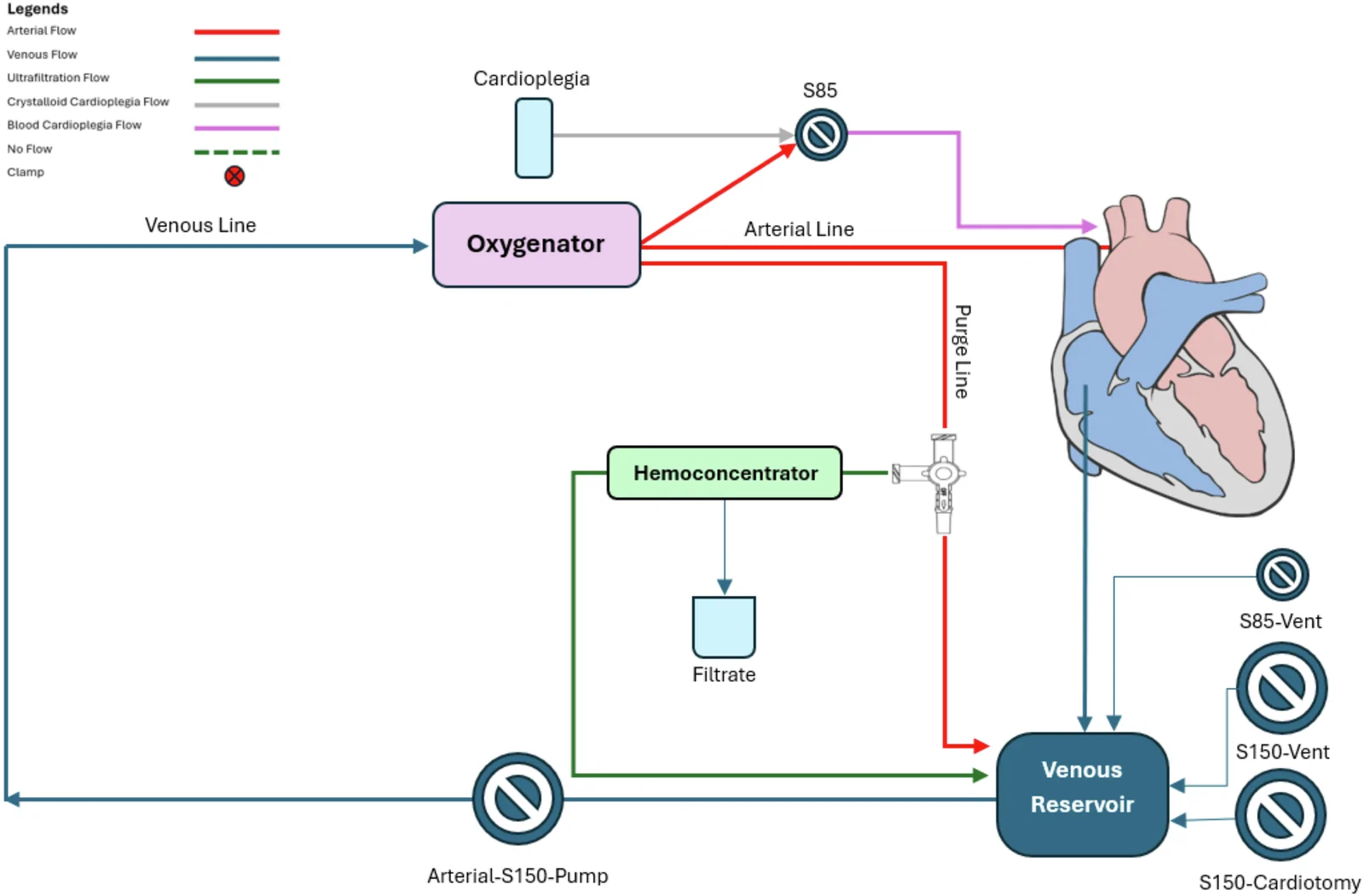
Conventional ultrafiltration (CUF): Hemofilter inflow connected to oxygenator purge line; outflow to venous reservoir.

**Figure 3:**
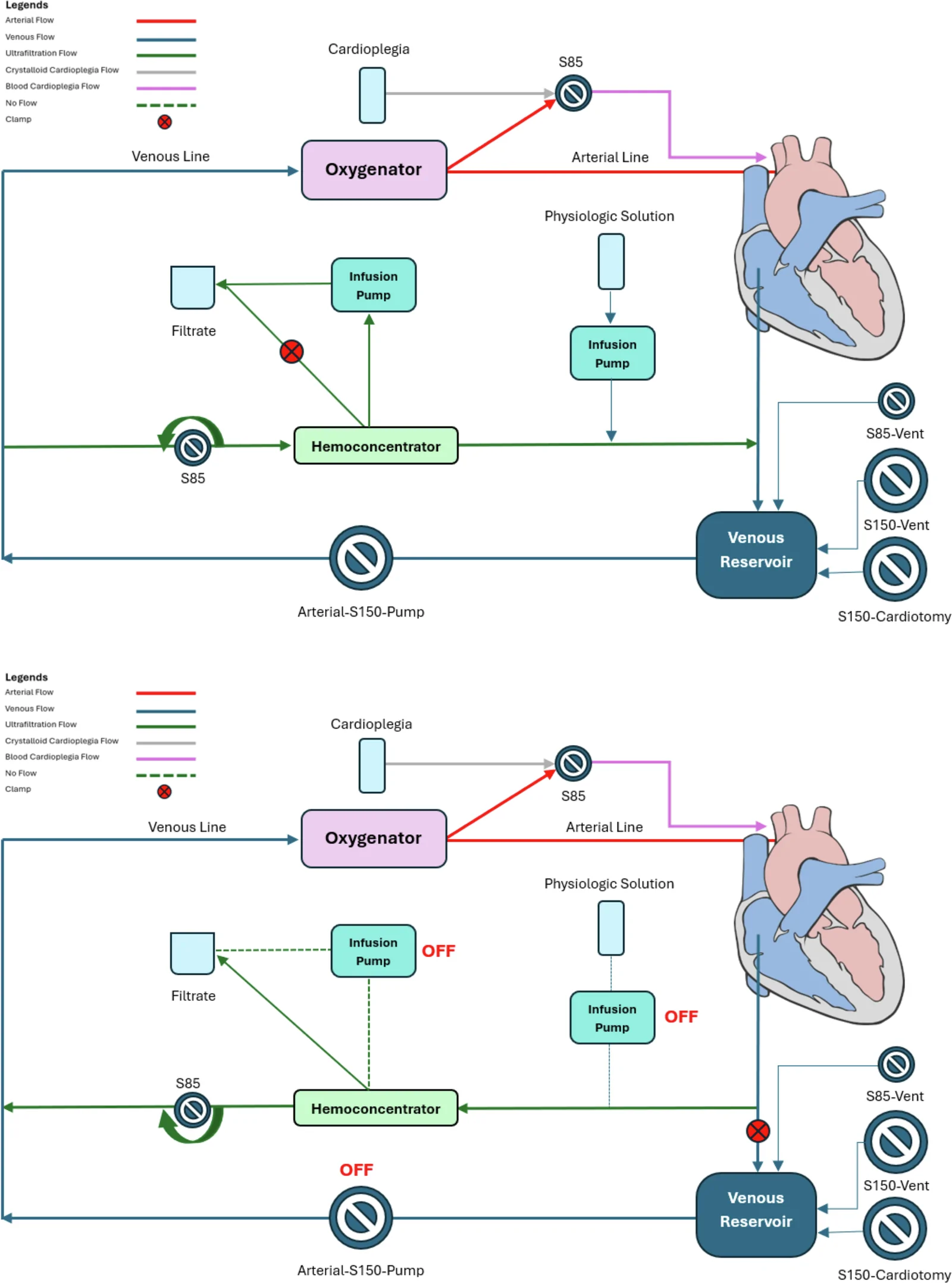
(A): Subzero-balance Ultrafiltration (SBUF) phase; Blood flow is directed in a veno-venous manner through an anti-clockwise rotation of the S85 roller pump. Infusion pumps effectively remove a specified volume of ultrafiltrate effluent and reinfuse a designated volume of physiological solution back into the venous reservoir. The clamp can be released to bypass the syringe pump regulation, allowing for high-volume ultrafiltration when necessary. (B): Simple modified Ultrafiltration (SMUF) phase; after the termination of the Continuous Perfusion Bypass (CPB), the S85 roller pump's direction is reversed to clockwise, and the stop-link is disengaged. Blood flow is redirected in a veno-arterial manner through the ultrafiltration circuit. During this process, the infusion pumps are deactivated, as indicated by the dotted lines. The S150 arterial pump can be activated to transfuse the patient if necessary.

**Figure 4:**
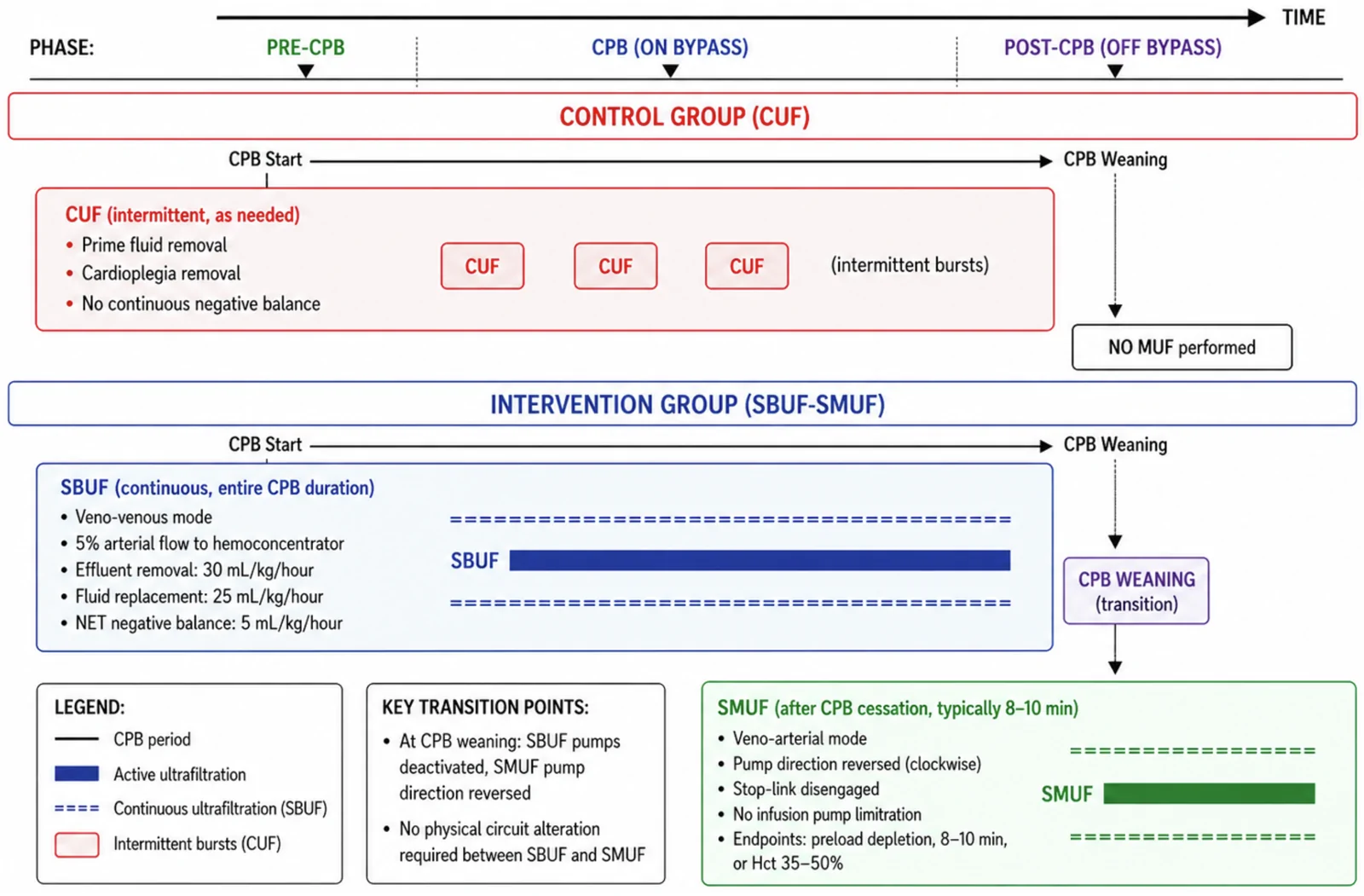
Control group (CUF): Intermittent CUF bursts during CPB; no post-bypass ultrafiltration. Intervention group (SBUF-SMUF): Continuous SBUF throughout entire CPB (net negative 5 mL/kg/h) followed by SMUF after CPB weaning (8–10 min, veno-arterial mode).

- Control Group (CUF): Hemofiltration was performed using the conventional ultrafiltration (CUF) technique. The hemofilter’s inflow line was connected to a three-way stopcock on the oxygenator's purge line, and the outflow was connected to the cardiotomy reservoir (Figure 2). No modified ultrafiltration (MUF) was performed after weaning.
- Intervention Group (SBUF-SMUF): In this group, a physiological solution consisting of 500 mL of 0.9% normal saline supplemented with 30 mL of 7.5% sodium bicarbonate was prepared and administered via a drip-count infusion pump prior to initiation of cardiopulmonary bypass (CPB). The hemoconcentrator (HC05) was incorporated into the circuit via a dedicated ultrafiltration loop. Blood was drained from the pre-reservoir segment of the venous line, passed through a dedicated roller pump (stop-linked to the arterial pump), and directed into the hemoconcentrator. During the Subzero-balance Ultrafiltration (SBUF) phase, the filtered blood was returned to the venous reservoir, establishing a venovenous ultrafiltration configuration, as illustrated in Figure 3A. SBUF was initiated at full CPB flow and maintained throughout the duration of CPB. The ultrafiltration flow rate was set at approximately 5% of the main arterial flow, with a target cardiac index of 2.4–3.0 L/min/m^2^. Two pumps were used concurrently: one for ultrafiltrate effluent removal (30 mL/kg/h) and another for replacement fluid infusion into the venous reservoir (25 mL/kg/h), resulting in a net fluid removal of 5 mL/kg/h. Following termination of CPB, the system was transitioned to simple modified ultrafiltration (SMUF). In this phase, the direction of the mini roller pump was reversed to clockwise, and the circuit configuration was modified such that blood was drained from the pre-reservoir venous line, passed through the hemoconcentrator, and returned to the arterial line proximal to the oxygenator (arterial pump outflow), thereby establishing a veno-arterial configuration, as depicted in Figure 3B. During SMUF, flow was maintained at approximately 5% of cardiac output, and infusion pumps were discontinued. Ultrafiltration was continued until one of the predefined endpoints was reached: inadequate preload (based on clinical assessment), a duration of 8–10 min, or achievement of a target hematocrit of 35–50% (Figure 4) [9].

### Equipment and disposable

The CPB circuit included a Liva Nova S5™ CPB System with S150 and S85 roller pumps, Terumo FX05 or FX15 oxygenators, and a Terumo Capiox Hemoconcentrator HC05. All tubing was non-miniaturized, phosphorylcholine-coated polyvinyl chloride.

### Blinding

The study was outcome-assessor-blinded and ICU-nurse-blinded. The perfusionist and operating room team could not be blinded due to the nature of the intervention. Patients who were mostly small children undergoing surgery were not aware of the specific procedures they received. Parents’ knowledge did not influence intraoperative or postoperative management. Additionally, ICU nurses responsible for the care and management of the child’s postoperative condition were unaware of the intervention done in the operating room. Perfusionists followed a standardized written protocol for each technique, reviewed in a pre-trial briefing session to reduce variability.

## Sample size

A total of 80 Patients were randomly divided into two groups with a statistical power of 90%.

## Anesthesia and surgery

Anesthesia for both groups included Midazolam, Fentanyl, Thiopental, and Cisatracurium. Cardioplegia was administered using a 20 mL/kg hyperkalemic solution containing 26 mEq/L potassium (Del Nido solution).

## Data collection

- **Preoperative Data**: Patient information was recorded for both the control and intervention groups prior to the initiation of the CPB circuit setup.
- **Postoperative Data**: Clinical and biochemical data were collected by the anesthesia team and ICU nurses following the patient's transfer to the ICU. Data was collected from the bypass chart, anesthesia chart, arterial blood gas (ABG) tests, and ICU records after discharge.

## Statistical analysis

Quantitative and qualitative data were analyzed using SPSS version 26. The statistical tests employed in the analysis included the independent *t*-test, Mann-Whitney *U* test, Chi-square test, Fisher's exact test, and Spearman correlation. Statistical significance was defined as a two-tailed *p*-Value of less than 0.05.

## Results

### Baseline and preoperative characteristics

A total of 80 pediatric patients undergoing cardiopulmonary bypass were randomized equally into two groups: the control group (CUF; *n* = 40) and the intervention group (SBUF-SMUF; *n* = 40) (Figure 1). Baseline demographic and preoperative characteristics were comparable between the groups (Table 1). The mean age was 2.82 ± 3.02 years in the CUF group and 3.00 ± 3.23 years in the SBUF-SMUF group (*p* = 0.973). Similarly, no significant differences were observed in weight (10.81 ± 5.95 vs. 12.08 ± 7.57 kg, *p* = 0.573), body surface area (0.50 ± 0.18 vs. 0.55 ± 0.25 m^2^, *p* = 0.540), or pre-bypass hematocrit (33.87 ± 4.94 vs. 33.06 ± 4.98%, *p* = 0.293).

**Table 1:** Demographic and preoperative characteristics.

Variables	Control group	Intervention group	*p*-Value
	(CUF) *N* = 40	(SBUF-SMUF) *N* = 40	
Age (y)	2.82 ± 3.02	3.00 ± 3.23	0.973
Weight (Kg)	10.81 ± 5.95	12.08 ± 7.57	0.573
BSA (m^2^)	0.50 ± 0.18	0.55 ± 0.25	0.540
Pre-bypass hct (%)	33.87 ± 4.94	33.06 ± 4.98	0.293

### Intraoperative and clinical characteristics

The distribution of surgical procedures was similar between the two groups (Table 2). The most common procedure was ventricular septal defect (VSD) repair in the CUF group (42.5%) and tetralogy of Fallot (TOF) repair in the SBUF-SMUF group (32.5%). Cardiopulmonary bypass (99.90 ± 32.74 vs. 111.87 ± 40.80 min, *p* = 0.137) and aortic cross-clamp times (65.75 ± 28.66 vs. 76.65 ± 33.69 min, *p* = 0.109) were not significantly different between groups.

**Table 2:** Clinical characteristics.

Variables	Control group	Intervention group	*p*-Value
	(CUF) *N* = 40	(SBUF-SMUF) *N* = 40	
Type of Procedure			
TOF	08	13	
VSD	17	13	
ASD	03	03	
AV canal	06	03	
DORV	04	01	
Others	02	07	
Bypass time	99.90 ± 32.74	111.87 ± 40.80	0.137
Clamp time	65.75 ± 28.66	76.65 ± 33.69	0.109
Fluid balance	192.1 ± 178.81	−105.0 ± 78.90	<0.001
Pre-bypass K^+^	3.52 ± 0.43	3.41 ± 0.38	0.242
10 min K^+^	4.58 ± 1.15	4.51 ± 0.88	0.729
30 min K^+^	4.51 ± 1.04	4.41 ± 1.03	0.637
60 min K^+^	4.61 ± 0.69	4.44 ± 0.75	0.376
90 min K^+^	4.50 ± 1.32	4.73 ± 0.81	0.787
120 min K^+^	4.01 ± 1.63	4.93 ± 1.12	0.286
Post bypass K^+^	3.82 ± 0.43	3.79 ± 0.58	0.599
Anes. PRBC Vol	261.00 ± 68.80	141.66 ± 70.172	<0.001
Post bypass FFP	10 (%)	02 (%)	0.020
ICU. PRBC Vol	261.00 ± 68.80	141.66 ± 70.17	<0.001
ICU. FFP	17	09	0.056
Extubating (days)	2.27 ± 2.37	1.05 ± 1.03	0.006

*p*-Value for distribution of procedure types between groups = 0.21 (Chi-square test).

A marked difference was observed in perioperative fluid balance, which was significantly positive in the CUF group (+192.1 ± 178.81 mL) compared with a negative balance in the SBUF-SMUF group (–105.0 ± 78.90 mL, *p* < 0.001). Potassium levels at various intraoperative time points were not significantly different between groups.

### Blood product utilization

The SBUF-SMUF group required significantly less intraoperative and postoperative blood product transfusion. The volume of packed red blood cells (PRBC) transfused intraoperatively was lower in the SBUF-SMUF group (141.66 ± 70.17 mL) compared with the CUF group (261.00 ± 68.80 mL, *p* < 0.001). Similarly, the need for postoperative fresh frozen plasma (FFP) was reduced in the SBUF-SMUF group (2% vs. 10%, *p* = 0.020). Postoperative PRBC transfusion requirements in the ICU remained significantly lower in the SBUF-SMUF group (141.66 ± 70.17 vs. 261.00 ± 68.80 mL, *p* < 0.001). Although ICU FFP use was lower in the SBUF-SMUF group (9 vs. 17 patients), this difference did not reach statistical significance (*p* = 0.056).

### Postoperative outcomes

Patients in the SBUF-SMUF group experienced earlier extubation compared with the CUF group (1.05 ± 1.03 vs. 2.27 ± 2.37 days, *p* = 0.006). Post-bypass hematocrit was significantly higher in the SBUF-SMUF group (31.2% vs. 27.3%, *p* = 0.001). No significant differences were observed in postoperative potassium levels between groups.

## Discussion

The types of surgeries in which different ultrafiltration techniques were performed were not significantly different between the intervention and control groups, with a *p*-Value = 0.21. VSD was the most common case with 17 cases in the control group. In the intervention group, VSD and TOF defects included 13 cases each, which, like the Bjornard statistical study in 2013, confirms the higher prevalence of VSD defects than any other CHD [10].

According to the instructions of performing the SBUF technique, net fluid removal of at least 5 mL/kg/h was targeted according to the weight (12 ± 7 kg) and duration of bypass (111 ± 40 min) in the intervention group, an average balance of −105 mL was achieved. This fluid balance depends solely on the volume of the crystalloid fluid, and the volume of transfused blood is independent of it.

In the control group, according to the CUF technique, continuous fluid administration was not required, and only prime fluid removal and cardioplegia removal were attempted. The average balance during bypass in this group was +192 mL, which is a sign of imposing more crystalloid fluids on the child. According to the Grist’s study in 2011, positive fluid balance, especially in infants under 6 kg, significantly increases the risk of death. Balances less than −40 mL/kg also increase the chance of mortality [11].

Potassium ions are among the most important ions monitored during and after heart surgery. In the intervention group, due to the replacement of a large volume of fluid and net fluid removal during the SMUF stage, the possibility of a dangerous reduction in potassium ions in the period after bypass was noted. It should be noted that the cardioplegia solution was hyperkalemic in both groups, and almost all children received a bag of blood product during bypass, which is the source of potassium. According to ABG reports, pre-bypass potassium level in both groups without a statistically significant difference was about 3.5 mmol / L. At different times during bypass, there were no significant differences. and post-bypass, the average potassium in the intervention group was 3.79 mmol / L, which is still away from the minimum level.

While performing the SMUF technique in the intervention group, anesthesia team support was essential for maintaining adequate Blood Pressure. In this phase of the ultrafiltration technique, increasing hematocrit and concentrating coagulation factors were attempted. The mean hematocrit post-bypass was 31.2% in the SBUF-SMUF group and 27.3% in the CUF group, with a *p*-Value = 0.001; this difference is quite significant. These results are in line with the findings of Niu, who performed combined hemofiltration with MUF in 2019, although hematocrit levels were higher in their intervention group [12].

In the intervention group, 12 children received packed red blood cell (PRBC) transfusions with an average volume of 141 mL per child. In the control group, 30 children received PRBC transfusions with an average volume of 261 mL per child. These differences in volume are both statistically and clinically significant.

Regarding immediate post-bypass FFP transfusions, 10 patients from the control group and 2 patients from the intervention group received 2 packs of FFP, with a significant difference between the groups (*p*-Value = 0.02). These two findings confirm the beneficial effect of the SBUF-SMUF technique on the storage of blood products, reducing the risk of blood-borne diseases and economic costs.

In the Stammers review study from 2016 to 2019, a decrease in urinary output following the use of the ZBUF technique in adult patients was emphasized [13]. Therefore, due to the use of continuous ultrafiltration in the SBUF-SMUF group, the rate of urinary output during bypass and up to 3 days in the ICU was compared between the two groups. There was no statistically or clinically significant difference between the groups at any time, and it can be stated that continuous and prolonged ultrafiltration was not a risk factor for the occurrence of AKI in the pediatric population.

During the ICU stay, 34 children in the intervention group with a mean volume of 141 mL and 40 children in the control group with a mean volume of 261 mL received homologous red blood cell product, which in terms of volume showed a significant difference with *p*-Value = < 0.005.

In terms of receiving FFP during ICU stay, 9 children in the intervention group and 17 children in the control group underwent FFP transfusion. Although the consumption of FFP in the control group was almost double, with the *p*-Value = 0.056, we can say that there is no statistically significant difference between them. Again, the efficiency of the SBUF-SMUF technique in storing a variety of blood products is remarkable.

Another variable that was chest tube output (mL) monitored and compared during the first 3 days of stay in the ICU was the amount of drainage of patients. As expected, the amount of bleeding and drainage gradually decreased in both groups, but the amount was not clinically and statistically different between the two groups. This finding was in contrast to Ziyaeifard's 2017 finding, that after performing a combination technique including MUF, they had reported a clinically significant decline in chest drainage of children [14].

In order to obtain an overview of the effect of the SBUF-SMUF technique on the improvement of pulmonary function after surgery, the patients' extubation time was also compared. Aligning with many previous studies, our findings were also promising in this regard. On average, patients in the SBUF-SMUF group were extubated approximately 24 h after entering the ICU, but patients in the CUF group needed a little more than 48 h to be extubated. This difference is also statistically significant with a *p*-Value = 0.006. As stated by Turkoz in a 2014 study, performing MUF by removing fluid reduces hydrostatic pressure and pulmonary edema, which increases lung capacity and decreases the alveolar-arterial gradient [15]. On the other hand, according to Bando's findings, by using the continuous ultrafiltration technique, inflammatory factors such as endothelin-1, which causes pulmonary vascular resistance, are also eliminated [16]. As a result, the improvement of pulmonary function can be confirmed by using this new combination technique.

Spearman’s test was used to examine the relationship between balance and extubation time, which was obtained in the correlation intervention group at 37%. But no significant correlation was observed in the control group.

Regarding the last variable, which is the length of ICU stay, no statistically significant difference was observed with *p*-Value = 0.33, and for both groups, it was about 5 days, which is in line with most studies related to hemofiltration [17].

### Limitations and important conceptual factors

We recognize that institutional practices regarding Continuous Ultrafiltration (CUF) can vary. At our center, CUF was primarily performed to remove prime volume and cardioplegia, rather than to achieve a net negative fluid balance. This approach may differ from other centers that utilize CUF more aggressively for volume removal. Therefore, our findings should be understood as a comparison between Standard Bypass and Standard MUF (SBUF-SMUF) against a more limited CUF strategy. We cannot rule out the possibility that combining CUF with MUF (CUF-MUF) could yield similar results. However, SBUF-SMUF provides continuous and controlled fluid removal during bypass (SBUF), followed by post-bypass concentration (SMUF). This method may offer advantages over CUF-MUF in terms of maintaining hemodynamic stability and avoiding steal syndrome. Future studies should directly compare SBUF-SMUF with CUF-MUF. It is important to note that sodium and calcium levels were not systematically analyzed in this study, which is a limitation given the bicarbonate-rich replacement fluid used in SBUF.

## Conclusion

Clinical and biochemical findings from this study indicate that the SBUF-SMUF combined ultrafiltration technique can be performed with conventional equipment, and if performed accurately and with proper monitoring, it is without complications. No apparent additional risk of oliguria or potassium imbalance was observed with the new technique compared to CUF. Its beneficial effects in reducing the consumption of blood products and the duration of intubation in children have been recognized. The SBUF-SMUF circuit provides fast, controlled fluid removal during bypass. The surgeon-independence of SMUF and avoidance of "steal syndrome" may encourage implementation and offer advantages over classical MUF. On the path to standardizing pediatric hemofiltration, the SBUF-SMUF technique, due to its ease of conduction and maximum benefits, has shown that it can be considered in future studies and clinical practices.

## Recommendation

Now that the efficacy, safety, and benefits of the SBUF-SMUF technique have been somewhat clarified, further research can investigate its effects on the concentration and excretion of specific coagulation and inflammatory agents. A direct comparison between SBUF-SMUF and CUF-MUF would be a valuable next step.

## Data Availability

The data from this study are available upon reasonable request from the corresponding author.
